# Characterization of microbial communities in flavors and fragrances during storage

**DOI:** 10.3389/fmicb.2025.1516594

**Published:** 2025-01-22

**Authors:** Yingjie Feng, Tingting Zhang, Jinchu Yang, Wenzhao Liu, Yongfeng Yang, Jihong Huang, Shen Huang, Zongcan Yang, Qianjin Liu, Wenchao Zheng, Qing Zhou

**Affiliations:** ^1^Technology Center, China Tobacco Henan Industrial Co., Ltd, Zhengzhou, China; ^2^State Key Laboratory of Crop Stress Adaptation and Improvement, College of Agriculture, Henan University, Kaifeng, China; ^3^College of Tobacco Science and Engineering, Zhengzhou University of Light Industry, Zhengzhou, China

**Keywords:** flavors and fragrances, whole genome shotgun (WGS), high-throughput sequencing, spoilage, GC–MS

## Abstract

Flavors and fragrances are essential for product quality, yet they are highly susceptible to contamination due to high moisture content and rich nutrients. This study investigates microbial growth, pH changes, volatile compound dynamics, and microbial community changes during the storage of flavors and fragrances. Results indicate that total viable counts (TVC) remained stable for the first three days but increased rapidly afterward, exceeding the acceptable limit of 5 log CFU/mL by day 7. The pH levels initially rose slightly, followed by a steady decline, which indicates spoilage progression. Gas chromatography–mass spectrometry (GC–MS) analysis revealed significant degradation of key aromatic compounds, such as 5-hydroxymethylfurfural (5-HMF), vanillin, and its derivative ethyl vanillin. Whole genome shotgun (WGS) sequencing demonstrated a marked increase in microbial community richness and diversity as storage progressed, with a notable shift in composition. Early storage stages were dominated by fungal species from the *Ascomycota* phylum, while later stages saw a rise in spoilage-associated bacteria, particularly from the *Firmicutes* and *Proteobacteria* phyla. Throughout the storage process, *Zygosaccharomyces* and its dominant species, *Zygosaccharomyces bailii*, remained prevalent, though their average relative abundance decreased from 81.26 to 32.29%. In addition, the bacterial species *Oceanobacillus sojae* and *Niallia nealsonii* showed significant increases in relative abundance, suggesting that bacteria were one of the key contributors to the spoilage of flavors and fragrances. Functional analysis based on the Kyoto Encyclopedia of Genes and Genomes (KEGG) database indicated a shift in metabolic pathways within the microbial community, with heightened metabolic activity correlating with spoilage. These findings provide valuable insights for improving storage methods and quality control of flavors and fragrances.

## 1 Introduction

Flavors and fragrances are intricate mixtures of aromatic compounds that provide distinctive and pleasant scents. In 2024, the global market for flavors and fragrances reached approximately $28.72 billion, with widespread applications across food, beverages, tobacco, perfumes, and cosmetics fields (Flavors and Fragrances Market, 2024; Adeboye et al., 2014; Zhu and Xiao, 2022). The flavor and aroma of food are critical determinants of consumer preferences, and even small additions of flavorings can significantly enhance product quality and customer satisfaction, ultimately driving consumption (Shukla et al., 2019).

Most food products naturally harbor bacteria unless they undergo specific sterilization (Bloomfield et al., 2023). Irradiation has been demonstrated to effectively inhibit the growth of harmful microorganisms, such as *Staphylococcus aureus* (Nortjé et al., 2006), *Salmonella* (Calle et al., 2021), and *Bacillus cereus* (Hong et al., 2008), without compromising food quality. According to Chinese national standards (GB/T 18526.4-2001), the total bacterial count in irradiated products should not exceed 1.5 × 10^4^ CFU/mL. However, due to the high moisture content and organic composition, flavors and fragrances are particularly susceptible to microbial proliferation, which poses storage challenges and leads to spoilage, resulting in economic losses and resource waste. Spoilage in flavors and fragrances typically results from oxidation from air exposure, the development of off-flavors and off-odors, as well as microbiological contamination (Koutsoumanis et al., 2021). Of these, microbiological spoilage is the most prevalent, with bacteria, yeasts, and molds playing major roles. Currently, flavor quality evaluation heavily relies on subjective sensory assessments, lacking objective microbial monitoring techniques.

In recent years, microbial spoilage has emerged as a major concern in the industry, with particular attention to the dynamics of spoilage bacteria during storage (Xia et al., 2023). Microorganisms are the primary contributors to spoilage in most seafood products. However, only a few specific microbial communities can proliferate extensively, ultimately leading to spoilage, which are referred to as specific spoilage organisms (SSOs) (Gram and Dalgaard, 2002). Understanding these spoilage microorganisms across various foods is crucial for controlling microbial growth and extending shelf life. Traditional molecular biology methods, such as polymerase chain reaction (PCR) and 16S rRNA gene analysis, have proven insufficient for comprehensive studies of microbial dynamics in food (Bekaert et al., 2015; Ezeokoli et al., 2016; Olofsson et al., 2007). High-throughput sequencing (HTS) technologies, such as the Illumina NovaSeq platform, have emerged as a powerful tool for accurately characterizing SSOs in food products. For example, *Shewanella* spp. is recognized as a dominant spoilage organism due to its significant amine metabolism capabilities (Zhang et al., 2022; Zhu et al., 2016). Furthermore, HTS has also been widely applied to examine the predominant spoilage microorganisms under various storage conditions. Studies have shown that *Staphylococcus* and *Serratia* are key spoilage genera in modified atmosphere and vacuum-packaged braised chicken, respectively (Lei et al., 2022). In Daokou braised chickens, predominant spoilage bacteria include *Enterococcus*, *Serratia*, *Vagococcus*, and *Lactobacillus* (Zhang et al., 2023), and *Pseudomonas* is recognized as the primary spoilage organism in Spanish mackerel (Zheng et al., 2020). HTS enables detailed analysis of dominant microbial characteristics, such as their aerobic or anaerobic nature, which can provide insights into strategies for extending shelf life (Zhu et al., 2021). Additionally, functional predictions based on the KEGG database can further elucidate the potential metabolic functions of microbial communities, highlighting their impact on spoilage mechanisms and the stability of flavors and fragrances.

Despite the application of HTS in studying SSOs across various products, research on the microorganisms responsible for spoilage in flavors and fragrances remains limited. This study aims to investigate the total viable count (TVC) and pH changes of flavors and fragrances during storage. Gas chromatography–mass spectrometry (GC–MS) is employed to analyze the effects of storage on volatile compounds. Furthermore, whole-genome sequencing (WGS) is utilized to examine microbial community diversity, composition, and function throughout storage, ultimately providing a theoretical foundation for extending the shelf life of flavors and fragrances.

## 2 Materials and methods

### 2.1 Sample collection and storage

Flavors and fragrances samples were provided by China Tobacco Henan Industrial Co., Ltd. To simulate typical production process, we opened fully packaged samples, took portions, and then resealed and transported them to the laboratory. In the lab, the samples were divided into two groups: three were stored at −80°C as the initial storage group (Group A), and the remaining three were stored at room temperature (25°C, 60% relative humidity) for 14 days as the end of storage group (Group B). Additionally, samples were collected on days 0, 3, 7, 10, 14, and 17 for total viable count (TVC) and pH values measurement. Volatile compounds analysis was conducted on samples collected on days 0, 7, and 14.

### 2.2 Total viable count (TVC) and pH measurement

TVC was measured according to the China National Food Safety Standard Method for Food Microbiological Examination (GB 4789.2-2022). Briefly, 25 mL of sample was mixed with 225 mL of sterile saline solution to prepare a 1:10 dilution. This mixture was then serially diluted in ten-fold increments. For each dilution, 1 mL was transferred to a sterile Petri dish, followed by the addition of 15 mL of plate count agar. After thorough mixing, the plates were incubated at 37°C for 48 h, and colony counts were expressed as CFU/mL.

For pH values measurements, a Mettler Toledo Seven2GO™ pH meter was used, following the manufacturer’s instructions. Measurements were taken at regular intervals to monitor pH changes throughout the storage period at room temperature.

### 2.3 Volatile compounds analysis

Volatile compounds in flavors and fragrances were analyzed using gas chromatography–mass spectrometry (GC–MS). The 2 mL sample of flavors and fragrances was placed in a centrifuge tube, followed by the addition of 10 mL PBS. The mixture was vortexed for 5 min at 2,500 rpm. Next, 10 mL of HPLC-grade acetonitrile and 50 μL of internal standard (2,3-dichlorotoluene, 0.2096 g/L) were added, and the mixture was vortexed for 20 min at 2500 rpm. After freezing the sample at −20°C for 30 min, 1.5 g NaCl and 6 g MgSO₄ were added followed by quick shaking. Then, 5 mL of HPLC-grade dichloromethane was added, vortexed for 10 min at 2,500 rpm, and centrifuged for 3 min at 8,000 rpm. The supernatant was collected, concentrated, filtered, and subsequently analyzed using GC–MS.

The chromatographic conditions were as follows: an HP-5MS capillary column (60 m × 250 μm × 0.25 μm) was used. The inlet temperature was set to 240°C, with a 1.0 μL injection volume and helium as the carrier gas at a flow rate of 1.0 mL/min in splitless mode. The temperature program was as follows: hold at 50°C for 4 min, increase by 3°C/min to 70°C and hold for 5 min, then increase by 2°C/min to 100°C and hold for 17 min, followed by an increase to 120°C (hold for 10 min), and finally increase by 6°C/min to 280°C.

For mass spectrometry, electron ionization (EI) was used with an electron energy of 70 eV. The solvent delay was set to 10 min, and full-scan detection was performed with a mass range of m/z 35–500. Compound identification was achieved by comparing the retention times with known flavors and fragrances components and using the NIST20 spectral library. Quantification was performed using the internal standard method for compounds with available standards, while those without standards were quantified based on relative peak areas (compound peak area to internal standard peak area).

### 2.4 Whole genome shotgun (WGS) analysis

Microbial genomic DNA was extracted using the OMEGA Mag-Bind Soil DNA Kit (M5635-02) following the manufacturer’s instructions. The extracted DNA was stored at −20°C. DNA quality and quantity were measured using agarose gel electrophoresis and a Qubit™ 4 Fluorometer. Metagenome shotgun sequencing libraries were prepared using the Illumina TruSeq Nano DNA LT Library Preparation Kit with an insert size of 400 bp. Sequencing was conducted on the Illumina NovaSeq platform (PE150) at Personal Biotechnology Co., Ltd. (Shanghai, China).

Raw sequencing reads were processed to generate high-quality reads. First, sequencing adapters were removed using Cutadapt (v1.2.1), followed by trimming of low-quality reads using the sliding-window algorithm in fastp. Taxonomic classifications of reads were performed using Kraken2 against a RefSeq-derived database (including archaea, bacteria, viruses, fungi, protozoans, metazoans, and plants). Reads assigned to metazoans and plants were excluded from downstream analysis. Alternatively, Kaiju with greedy-5 mode was used against an NR-derived protein database including similar taxa.

Assembly of quality-filtered reads was conducted with Megahit (v1.1.2) using meta-large preset parameters. Contigs longer than 300 bp were clustered using mmseqs2 with an identity threshold of 95 and 90% coverage of the shorter contig. Taxonomic assignment was performed by aligning contigs against the NCBI-NT database using mmseqs2 with the taxonomy mode. Contigs assigned to plants or metazoans were excluded. Gene prediction was performed using MetaGeneMark, and coding sequences (CDS) were clustered using mmseqs2 with 90% identity and coverage.

To assess gene abundances, high-quality reads were mapped to predicted genes using Salmon in quasi-mapping mode. Abundance was normalized using CPM (copies per kilobase per million mapped reads). Functional annotation was performed using mmseqs2 against Kyoto Encyclopedia of Genes and Genomes (KEGG) database. Linear discriminant analysis effect size (LEfSe) was applied to detect differentially abundant taxa and functions across groups. Beta diversity analysis was conducted using Bray-Curtis distance metrics and visualized through principal coordinate analysis (PCoA).

## 3 Results and discussion

### 3.1 Total viable counts and pH changes during storage

**Figure 1:**
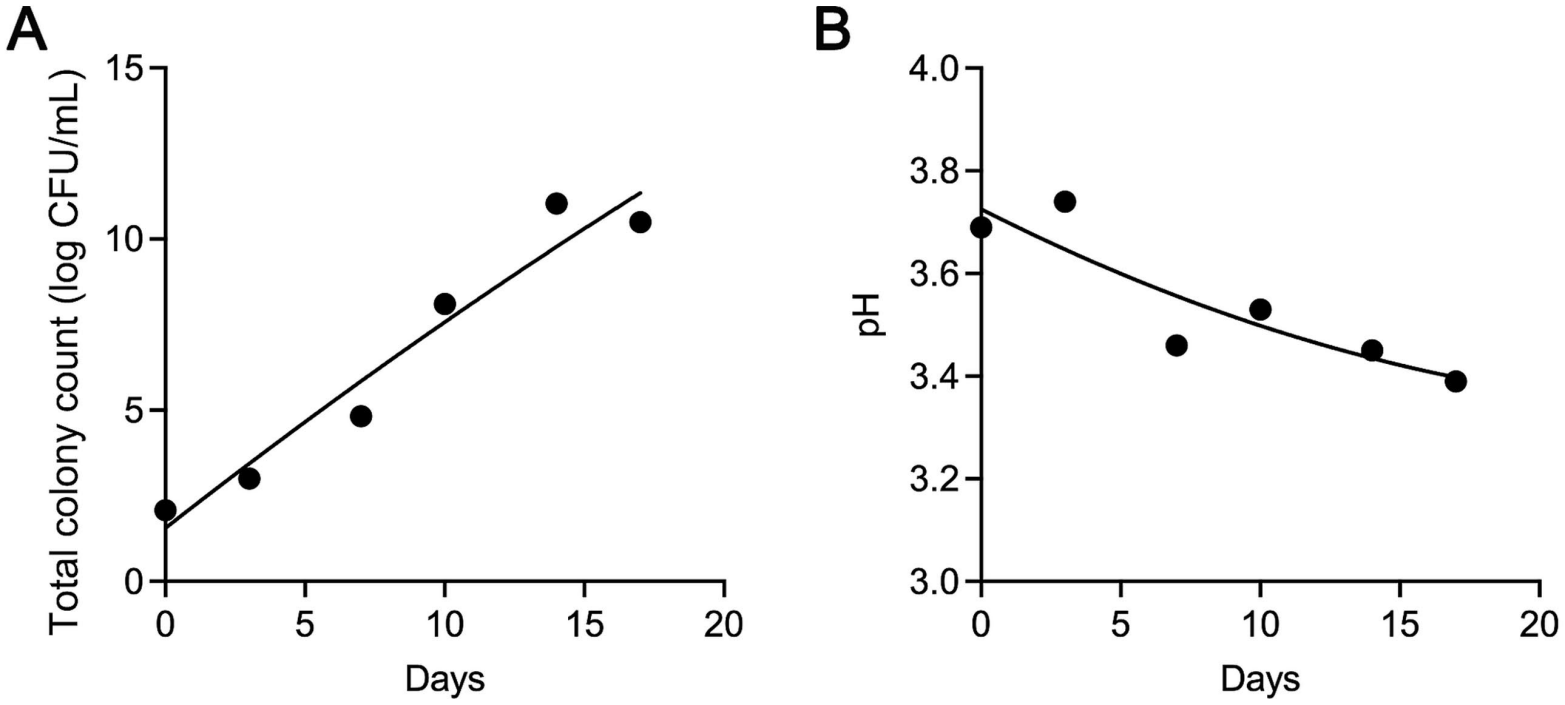
Changes in total viable count (TVC) **(A)** and pH values **(B)** during the storage of flavors and fragrances.

Microbial activity significantly influences the shelf life of flavors and fragrances, with total viable counts (TVC) serving as a direct indicator of microbial growth during storage. As shown in Figure 1A, the initial TVC was 2 log CFU/mL, indicating minimal contamination during production and packaging. For the first 3 days, TVC remained stable at 2–3 log CFU/mL, with no significant changes observed. However, after day 3, a rapid increase occurred, with TVC reaching 11 log CFU/mL by day 14. Beyond this point, microbial growth plateaued, likely due to the depletion of available organic nutrients, which limited further proliferation. According to the National Standard of China (GB 30616-2020), the acceptable TVC level is 5 log CFU/mL. Based on this standard, the sample remained within acceptable limits for up to 7 days.

In addition to TVC, monitoring pH values changes also provides valuable insights into spoilage progression. As shown in Figure 1B, the pH rose slightly from 3.69 to 3.74 early in storage, followed by a steady decline to 3.39 by day 14. This initial rise may be due to the production of alkaline compounds during early microbial metabolism. As storage continued, the accumulation of acidic by-products, particularly from acid-producing bacteria, became more pronounced, leading to a decline in pH as spoilage advanced. This trend is consistent with findings by Zhang et al. (2023), who observed a similar pH pattern in the spoilage of braised chicken, with an initial increase followed by a decline.

### 3.2 Volatile components analysis

Aroma loss significantly impacts the quality of flavors and fragrances during storage (Obenland et al., 2011). In this study, gas chromatography–mass spectrometry (GC–MS) was employed to analyze dynamic changes in volatile compounds over time. Volatile compounds with initial concentrations of no less than 1 μg/mL were listed in Table 1. The internal standard, 2,3-dichlorotoluene, showed consistent concentrations across three measurements, confirming the instrument’s reproducibility.

**Table 1:** GC–MS analysis of volatile compounds.

Kinds	CAS No.	Compound name	0 Day	7 Days	14 Days	Decrease
			mg/mL	mg/mL	mg/mL	%
Standard	32768-54-0	2,3-Dichlorotoluene	0.0115	0.0115	0.0115	0.00
Aldehyde	121-32-4	Ethyl vanillin	0.0038	–	–	100.00
Aldehyde	67-47-0	5-Hydroxymethylfurfural (5-HMF)	0.0252	0.0029	–	100.00
Aldehyde	121-33-5	Vanillin	0.0155	–	–	100.00
Ester	103-48-0	Phenethyl isobutyrate	0.0059	0.0016	0.0013	77.97
Ester	626-11-9	DL-Malic acid diethyl ester	0.001	0.001	0.0007	30.00
Ester	103-45-7	Phenethyl acetate	0.0091	0.0074	0.0074	18.68
Ester	623-50-7	Ethyl glycolate	0.003	0.0028	0.0027	10.00
Ester	10094-34-5	Benzyldimethylcarbinyl butyrate	0.0027	0.0016	0.0025	7.41
Ester	120-51-4	Benzyl benzoate	0.0026	0.0015	0.0031	−19.23
Ester	687-47-8	Ethyl L(−)-lactate	0.0028	0.003	0.0032	−14.29
Ester	28664-35-9	Sotolon	0.003	0.003	0.0034	−13.33
Ester	117-81-7	Bis(2-ethylhexyl) phthalate	0.0024	0.0024	0.0025	−4.17
Ester	7619-08-1	Ethyl linoleate	–	0.001	0.0016	
Ester	1962-75-0	Dibutyl terephthalate	0.0094	0.0091	0.0096	−2.13
Ester	627-69-0	2-Hydroxypropylacetate	–	0.0014	0.002	
Phenol	119-47-1	2,2′-Methylenebis(6-tert-butyl-4-methylphenol)	0.0131	0.0044	0.0051	61.07
Phenol	96-76-4	2,4-Di-tert-butylphenol	0.0011	0.0009	0.0009	18.18
Phenol	4940-11-8	Ethyl maltol	0.0067	0.0067	0.0075	−11.94
Acid	57-10-3	Palmitic acid	0.0014	–	–	100.00
Acid	65-85-0	Benzoic acid	0.0011	0.0019	0.0024	−118.18
Acid	110-44-1	Sorbic acid	0.0032	0.0037	0.0021	34.38
Alcohol	60-12-8	Phenethyl alcohol	0.1189	0.1261	0.1363	−14.63
Alcohol	1883-75-6	2,5-Furandimethanol	–	0.0029	0.0068	
Alcohol	498-00-0	Vanillyl alcohol	–	–	0.0102	
Alcohol	100-51-6	Benzyl alcohol	0.3525	0.3677	0.3711	−5.28
Ketone	3658-77-3	Furaneol	0.0443	0.0516	0.0617	−39.28
Ketone	10230-62-3	2,4-Dihydroxy-2,5-dimethyl-3(2H)-furan-3-one	0.0029	0.0028	0.0019	34.48
Ketone	95338-37-7	2,4,4-Trimethoxy-2,5-cyclohexadiene-1-one	0.0015	–	–	100.00
Ketone	430-51-3	Fluoroacetone	–	0.1097	0.4478	

“–” not detected.

A total of 3 aldehydes, 12 esters, 3 phenols, 3 acids, 4 alcohols, and 4 ketones were identified. Among the aldehydes, 5-hydroxymethylfurfural (5-HMF), vanillin, and its derivative ethyl vanillin are notable aroma contributors. However, these compounds were not detected in the later stages of storage. Vanillin can undergo biotransformation into vanillic acid and vanillyl alcohol, facilitated by microorganisms like *Saccharomyces* or *Cystobasidium larynges* (Rönnander et al., 2018). By day 7, vanillin had completely degraded, while vanillyl alcohol, initially undetectable, increased to 0.0102 mg/mL by day 14. This suggested that vanillyl alcohol might be the degradation product of vanillin. Both vanillin and ethyl vanillin are widely used flavoring agents with a characteristic rich vanilla scent, with ethyl vanillin providing a more intense and long-lasting aroma. Notably, in addition to their aromatic properties, these compounds have demonstrated antimicrobial activity (Fitzgerald et al., 2003). Vanillin, for instance, has been shown to inhibit the growth of bacteria and fungi such as *Escherichia coli* and *Saccharomyces cerevisiae*, and to exert cytotoxic effects on mammalian cells (Adeboye et al., 2014; Bezerra et al., 2016; Cheng et al., 2007). Therefore, the presence of vanillin in samples contributes not only to aroma but also to the preservation of flavors and fragrances. Its degradation diminishes both the aroma profile and the preservative potential.

Ester compounds play a pivotal role in contributing fruity, floral, or sweet aromas, making them essential for enhancing the complexity and longevity of fragrances. For example, phenethyl isobutyrate and phenethyl acetate are widely used in fine perfumes, shampoos, soaps, and non-cosmetic products such as detergents and household cleaners (McGinty et al., 2012a; McGinty et al., 2012b). Sotolon is another significant compound, recognized for contributing to the characteristic roasted chicory aroma (Wu and Cadwallader, 2019). Additionally, palmarosa essential oil contains several key aromatic components, with benzyl benzoate being one of the most significant (Kou et al., 2023). In the later stages of storage, a noticeable decline in the concentrations of several esters, including phenethyl isobutyrate, DL-malic acid diethyl ester, phenethyl acetate, ethyl glycolate, and benzyldimethylcarbinyl butyrate, was observed. Notably, the concentration of phenethyl isobutyrate decreased by 77.97%. Conversely, some esters remained relatively stable or even showed slight increases, such as benzyl benzoate, ethyl L(-)-lactate, sotolon, bis (2-ethylhexyl) phthalate, ethyl linoleate, dibutyl terephthalate, and 2-hydroxypropylacetate. This variation is likely due to complex biochemical reactions that occur during storage, leading to the production of these compounds.

Sorbic acid, along with its sodium, potassium, and calcium salts, is often used in food preservation for its antifungal and antibacterial properties, working by disrupting microbial metabolism (Brul and Coote, 1999; de Jesus et al., 2021). When combined with vanillin, potassium sorbate has been shown to have a synergistic preservative effect (Kimani et al., 2023; Matamoros-León et al., 1999). In this study, a decline in sorbic acid concentration was also observed in the later stages of storage, further reducing its preservative efficacy. Whether sorbic acid and vanillin exhibit a similar synergistic effect in this study remains to be confirmed through further experimentation. Additionally, with prolonged storage, fluoroacetone levels were detected to gradually increase, raising concerns about the safety and quality of the products.

### 3.3 Whole genome shotgun (WGS) analysis

#### 3.3.1 Diversity analysis

**Figure 2:**
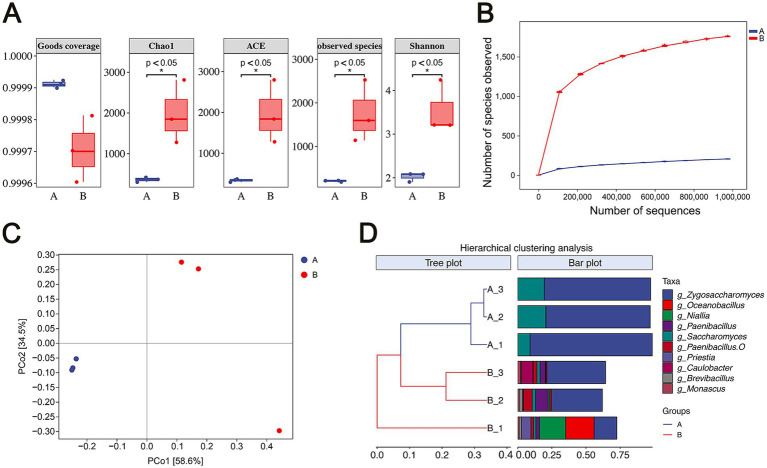
Analysis of microbial community diversity. **(A)** Alpha diversity of microbial communities. **(B)** Rarefaction curves of microbial communities. **(C)** Principal coordinate analysis (PCoA) of microbial communities based on Bray-Curtis distances. The horizontal and vertical axes represent relative distances without specific practical meaning. **(D)** Hierarchical cluster analysis (HCA) of microbial communities. In panels **B**, **C**, and **D**, Group A represents the early stage of storage, and Group B represents the end of storage.

The microbial diversity in flavors and fragrances during storage was analyzed using high-throughput sequencing. Alpha diversity, which assesses microbial richness and diversity within individual samples, was evaluated. As shown in the boxplot in Figure 2A, the Goods coverage index for all six samples exceeded 0.999, indicating that the sequencing depth was sufficient to capture the microbial diversity in each sample. Species richness was assessed using the Chao1, ACE, and observed species indices. By the end of storage (Group B), these indices were significantly higher compared to those at the early storage stage (Group A) (*p* < 0.05), demonstrating a substantial increase in microbial richness over time. Community diversity, measured by the Shannon index, also significantly increased in Group B compared to Group A, suggesting that the microbial community had become more diverse as storage progressed. The rarefaction curve based on the observed species showed a clear trend toward a saturation plateau, confirming that the sequencing adequately reflected community richness, with Group B showing greater diversity (Figure 2B).

Further analysis of beta diversity revealed significant differences in microbial community structure between the two groups. Principal Coordinates Analysis (PCoA), based on Bray-Curtis distances, indicated that the first principal component (PCo1) accounted for 58.6% of the total variation, while the second component (PCo2) explained 34.5%. The two groups were separated along the PCo1 axis, illustrating a distinct variation in community structure between them (Figure 2C). Hierarchical clustering analysis (HCA), based on Bray-Curtis distances and the top 10 genera by average relative abundance, depicted the similarity among the samples as a dendrogram, with Group A and Group B forming distinct clusters (Figure 2D). The PCoA and HCA results confirmed that the microbial community structures in Group A and Group B were distinctly different.

#### 3.3.2 Taxonomic composition of microbial communities

**Figure 3:**
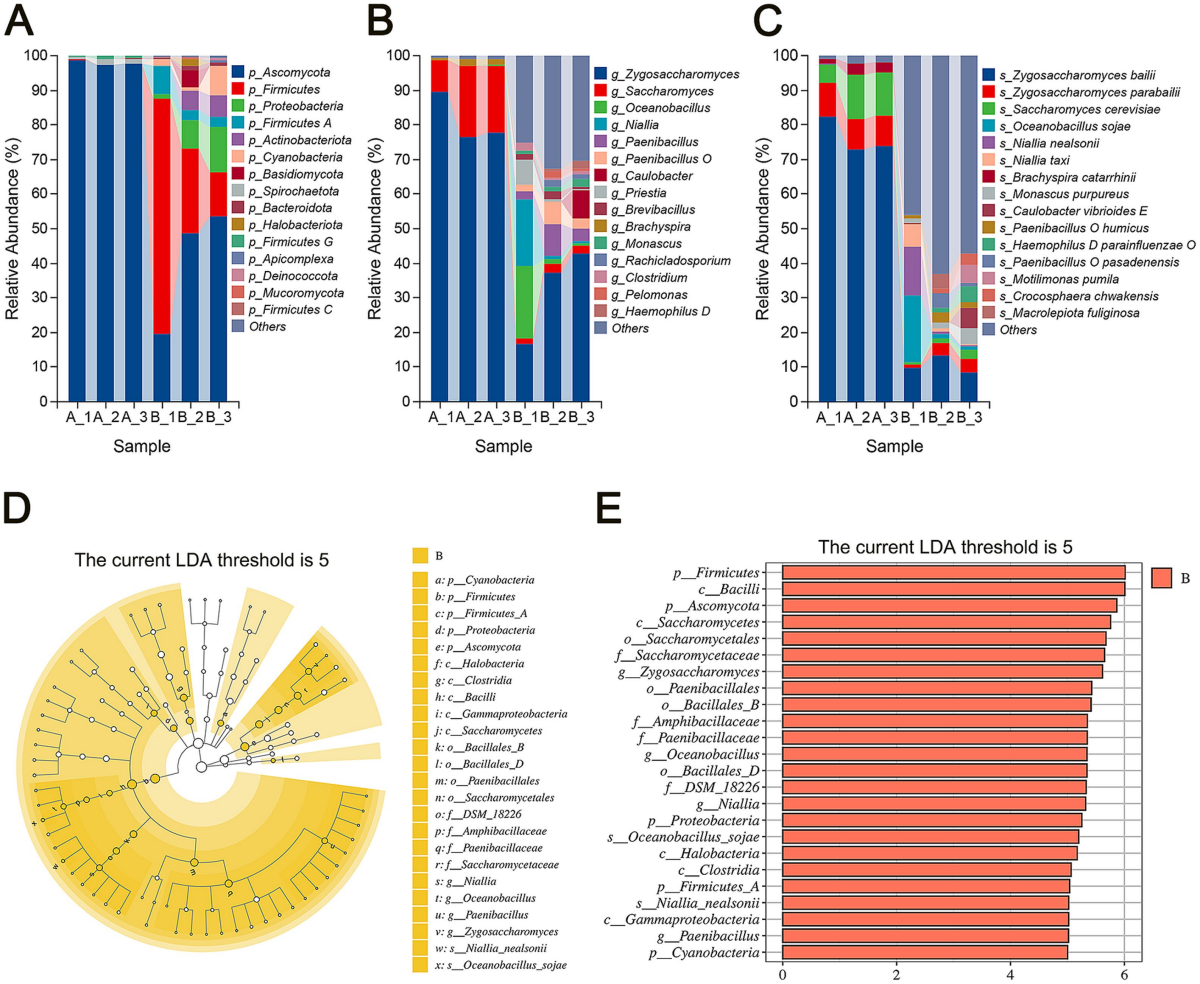
Composition analysis of microbial communities. **(A)** Relative abundance of the top 15 microbial phyla. **(B)** Relative abundance of the top 15 microbial genera. **(C)** Relative abundance of the top 15 microbial species. **(D)** Cladogram showing biomarkers identified through linear discriminant analysis (LDA) with a threshold score of 5. **(E)** LDA of significantly different microbial taxa with a threshold score of 5.

To investigate the impact of storage on microbial communities within flavors and fragrances, we conducted species annotation analysis across various taxonomic levels for both sample groups. The results show the top 15 microbial taxa by average relative abundance at the phylum and genus levels (Figures 3A,B).

At the phylum level (Figure 3A), *Ascomycota* initially dominated the microbial community in the early storage stage (Group A), with an average relative abundance of 97.86%, while other phyla had abundances below 1%. By the end of storage (Group B), microbial diversity increased significantly. Although *Ascomycota* remained the most prevalent phylum, its relative abundance decreased to 40.60%. Meanwhile, *Firmicutes* increased from 0.098% to 35.14%, *Proteobacteria* from 0.01% to 7.54%, and *Firmicutes A* from 0.03% to 4.72%. These shifts indicate a compositional change in the microbial community over time, with bacteria such as *Firmicutes* and *Proteobacteria* emerging as dominant by the end of storage. *Firmicutes* and *Proteobacteria* are commonly associated with spoilage in various foods, including fermented sausages (Zhang et al., 2021), braised chicken (Lei et al., 2022) and ready-to-eat chicken meat (Qiu et al., 2022).

At the genus level (Figure 3B), *Zygosaccharomyces* and *Saccharomyces* were predominant in Group A, with average relative abundances of 81.26% and 16.31%, respectively. In Group B, genera with average relative abundances exceeding 5% included *Zygosaccharomyces* (32.29%), *Oceanobacillus* (7.59%), *Niallia* (6.89%), and *Paenibacillus* (5.21%). Among the top 15 genera, fungal genera including *Zygosaccharomyces*, *Saccharomyces*, *Monascus*, and *Rachicladosporium* showed a marked decrease from 97.57% in the early stage to 37.06% by the end of storage. Both *Zygosaccharomyces* and *Saccharomyces* belong to the *Ascomycota* phylum and the *Saccharomycetaceae* family, and are well-known for their ability to thrive in extreme conditions, such as high sugar and salt concentrations. This resilience makes them common contaminants in products with high osmotic pressure, like syrups and soy sauce. The rise of bacterial genera such as *Oceanobacillus*, *Niallia*, and *Paenibacillus* suggests that these bacteria are significant contributors to spoilage during storage.

At the species level (Figure 3C), *Zygosaccharomyces bailii*, *Saccharomyces cerevisiae*, *Zygosaccharomyces parabailii*, and *Brachyspira catarrhinii* accounted for nearly all microbial populations in Group A. Among them, the fungal species *Zygosaccharomyces bailii* was dominant, with an average relative abundance of 76.40%. As storage progressed, the relative abundance of *Zygosaccharomyces bailii* decreased significantly to 10.57%, although it remained the predominant species. In contrast, the bacterial species *Oceanobacillus sojae* and *Niallia nealsonii* showed substantial increases, with their average relative abundances rising from 0.05% to 7.28% and from 0.03% to 5.00%, respectively.

We used Linear Discriminant Analysis Effect Size (LEfSe) to identify microbial biomarkers that differed significantly between the two groups (Zhu et al., 2021). A higher Linear Discriminant Analysis (LDA) score indicates a greater contribution of a species to the difference between groups. Using a Wilcoxon test and an LDA score threshold of 5, five phyla were found with higher relative abundances in Group B: *Cyanobacteria*, *Firmicutes*, *Firmicutes A*, *Proteobacteria*, and *Ascomycota* (Figures 3D,E). Among these, only *Ascomycota* is fungal, while the others are bacterial.

#### 3.3.3 Functional analysis of microbial communities

**Figure 4:**
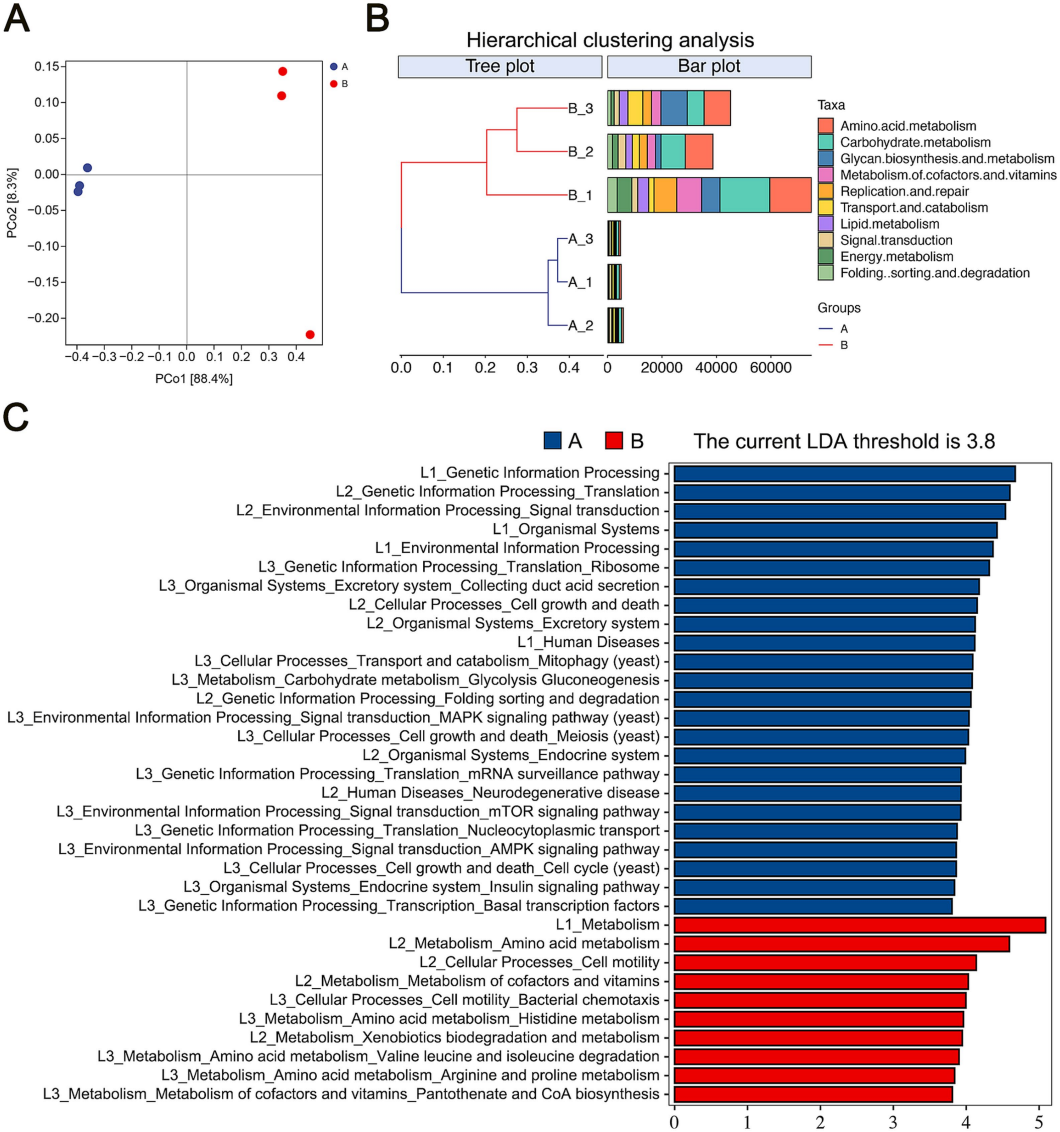
Functional analysis of microbial communities. **(A)** Beta diversity of microbial community functions, presented through PCoA. **(B)** HCA of microbial functions based on Bray-Curtis distances. **(C)** LDA of significantly different functions with a threshold score of 3.8.

We analyzed the functional differences between samples based on Bray-Curtis distance. Principal Coordinate Analysis (PCoA) indicated that the first axis (PCo1) explained 88.4% of the total variance, while the second axis (PCo2) accounted for 8.3%, separating the two sample groups along the PCo1 axis (Figure 4A). Hierarchical clustering analysis (HCA), using Bray-Curtis distances and KEGG Level 2 categories, also confirmed distinct functional differences between the groups, with Group A and Group B forming separate clusters (Figure 4B).

Further functional analysis via LEfSe, using an LDA score threshold of 3.8, identified significant KEGG pathway biomarkers between the groups. Group A was enriched in pathways related to genetic information processing, organismal systems, and human diseases at KEGG Level 1. In contrast, Group B was enriched in metabolism, suggesting that microbial metabolic activity is a primary driver of spoilage in flavors and fragrances during storage. Detailed analysis of Group B’s KEGG Level 2 pathways identified significant biomarkers in amino acid metabolism, metabolism of cofactors and vitamins, and xenobiotics biodegradation and metabolism. Additionally, cell motility emerged as a key functional biomarker for Group B (Figure 4C). These findings emphasize the functional shifts in microbial communities during storage, with metabolic activity playing a crucial role in the spoilage of flavors and fragrances.

## 4 Conclusion

This study investigated the relationship between microbial activity, volatile compound degradation, and the quality of flavors and fragrances during storage. The findings indicated that microbial growth, evidenced by increasing total viable counts (TVC) and a consistent decline in pH, significantly affected product quality. By day 7, TVC levels exceeded the acceptable limit, indicating spoilage. Simultaneously, key volatile compounds, such as ethyl vanillin, 5-hydroxymethylfurfural (5-HMF), and vanillin, degraded over time, resulting in diminished aroma and reduced preservative efficacy.

Metagenomic analysis revealed a notable increase in microbial richness and diversity, with a significant shift in community structure as storage progressed. While fungi dominated the microbial community during the early stages of storage, bacterial relative abundance increased substantially in the later stages. Functional analysis further confirmed that microbial metabolic activity played a critical role in driving spoilage, highlighting the importance of controlling microbial metabolism to extend the shelf life of flavors and fragrances. Although yeasts are generally considered beneficial microorganisms, certain spoilage yeasts, particularly those belonging to the *Zygosaccharomyces* genus, are major contaminants in the food and beverage industry (Fleet, 2011). In this study, *Zygosaccharomyces bailii* remained the dominant species throughout storage. This yeast is recognized as one of the most aggressive spoilage microorganisms, frequently contaminating products such as wine, fruit juices, carbonated soft drinks, and canned foods, especially those with high sugar content (Kuanyshev et al., 2017). Its high resistance to preservatives further complicates contamination control (Karaman et al., 2016). Interestingly, studies have shown that mint essential oil exhibits significant anti-yeast activity against *Zygosaccharomyces bailii* in apple juice. However, the combination of sodium benzoate and mint essential oil unexpectedly promoted *Zygosaccharomyce bailii* growth, demonstrating an antagonistic effect (Karaman et al., 2016). Additionally, some commercially available fermentates have shown inhibitory effects on *Zygosaccharomyce bailii*. For instance, a 1% concentration of Polyfence™ 35TD, a blend of cultured citrus extracts, significantly inhibited *Zygosaccharomyce bailii* and could serve as a potential replacement for potassium sorbate in dressings without affecting sensory quality (Samapundo et al., 2017).

In summary, this study provides valuable insights into the biological mechanisms underlying spoilage in flavors and fragrances during storage. It offers a theoretical foundation for developing strategies to prevent spoilage and extend product shelf life. Future research should focus on inhibiting the growth of dominant microbial species identified through high-throughput sequencing to improve product stability and longevity.

## Data Availability

The raw sequencing data of microbial communities related to flavors and fragrances have been deposited in the NCBI Sequence Read Archive (SRA) under BioProject accession number: PRJNA1191206.
